# Supplementary material for: Structural Changes and Astrocyte Response of the Lateral Geniculate Nucleus in a Ferret Model of Ocular Hypertension
Source: Int J Mol Sci. 2020 Feb 17;21(4):1339. doi: 10.3390/ijms21041339 (PMC7072923; doi:10.3390/ijms21041339)
Supplement: Supplementary file 1 [file ijms-21-01339-s001.pdf]

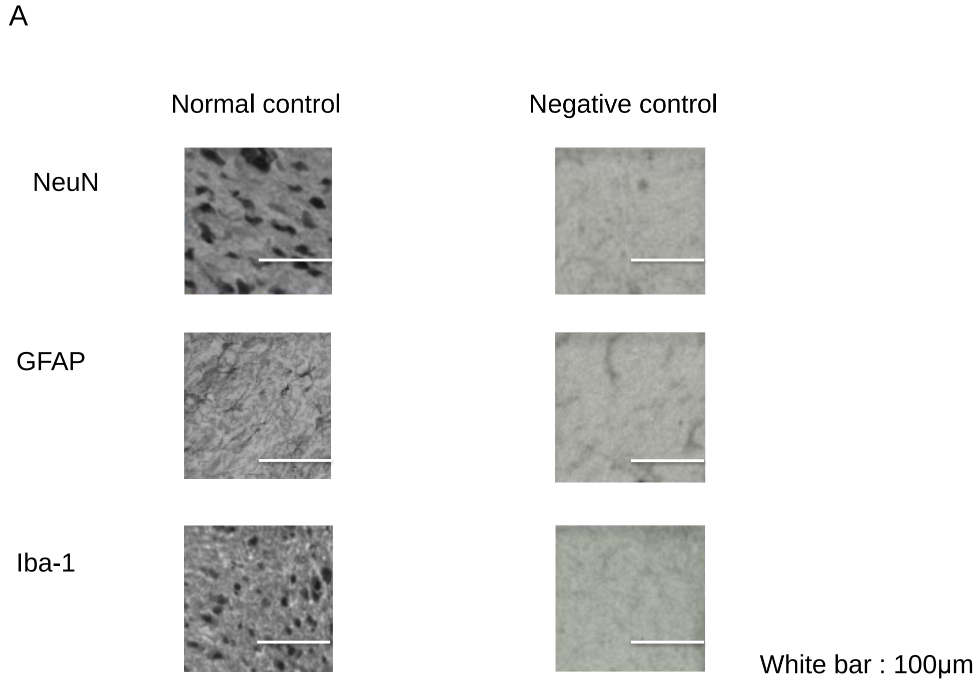

Supplemental data 1. Immunohistological images of neuroglial cells and glial cells in LGN of the control ferret. In the normal control, NeuN, astrocytes and microglia was stained well using 1st and 2nd antibodies. In the negative control, NeuN, astrocytes and microglia was not stained using only 2nd antibodies without 1st antibodies. (200 x 200 μm square). (white bar = 100 μm).
